# Subspace-based technique for speckle noise reduction in ultrasound images

**DOI:** 10.1186/1475-925X-13-154

**Published:** 2014-11-25

**Authors:** Norashikin Yahya, Nidal S Kamel, Aamir S Malik

**Affiliations:** Centre for Intelligent Signal and Imaging Research (CISIR), Universiti Teknologi Petronas, Bandar Seri Iskandar, Perak, Malaysia

**Keywords:** Speckle reduction, Denoising, Signal subspace, USDSAI

## Abstract

**Background and purpose:**

Ultrasound imaging is a very essential technique in medical diagnosis due to its being safe, economical and non-invasive nature. Despite its popularity, the US images, however, are corrupted with speckle noise, which reduces US images qualities, hampering image interpretation and processing stage. Hence, there are many efforts made by researches to formulate various despeckling methods for speckle reduction in US images.

**Methods:**

In this paper, a subspace-based speckle reduction technique in ultrasound images is proposed. The fundamental principle of subspace-based despeckling technique is to convert multiplicative speckle noise into additive via logarithmic transformation, then to decompose the vector space of the noisy image into signal and noise subspaces. Image enhancement is achieved by nulling the noise subspace and estimating the clean image from the remaining signal subspace. Linear estimation of the clean image is derived by minimizing image distortion while maintaining the residual noise energy below some given threshold. The real US data for validation purposes were acquired under the IRB protocol (200210851-7) at the University of California Davis, which is also consistent with NIH requirements.

**Results:**

Experiments are carried out using a synthetically generated B-mode ultrasound image, a computer generated cyst image and real ultrasound images. The performance of the proposed technique is compared with Lee, homomorphic wavelet and squeeze box filter (SBF) in terms of noise variance reduction, mean preservation, texture preservation and ultrasound despeckling assessment index (USDSAI). The results indicate better noise reduction capability with the simulated images by the SDC than Lee, Wavelet and SBF in addition to less blurry effect. With the real case scenario, the SDC, Lee, Wavelet and SBF are tested with images obtained from raw radio frequency (RF) data. Results generated using real US data indicate that, in addition to good contrast enhancement, the autocorrelation results shows better preservation of image texture by SDC than Lee, Wavelet and SBF.

**Conclusion:**

In general, the performance of the SDC filter is better than Lee, Wavelet and SBF in terms of noise reduction, improvement in image contrast and preservation of the autocorrelation profiles. Furthermore, the filter required less computational time compared to Lee, Wavelet and SBF, which indicates its suitability for real time application.

## Introduction

Ultrasound (US) imaging is one of the most commonly used medical imaging due for diagnostic purposes to its many advantages such as portability, the noninvasive nature, relatively low cost and presents no radiation risk to patient. These features have made the US imaging as the most prevalent diagnostic tool for health practitioners over other more sophisticated imaging techniques such as CT scan, MRI or PET. Unfortunately, like SAR, US images exhibit a speckle pattern and its statistical model is identical to single-look SAR amplitude signals. Speckle in ultrasound has adverse effect in such a way it causes reduction in image contrast resolution. In [1], Bamber and Daft show that speckle in US images cause reduction of lesion detectability by approximately a factor of eight.

An US machine works by introducing into the body of interest a low-energy pulse of sound with frequencies typically between 3 and 30MHz by a transducer probe that touches the patients’ skin surface. Upon travelling through the body tissue, some of the pulses get attenuated while some small portion of the pulse energy are scattered back to the probe. The scattered pulse is then received by the same probe to produce echo signals which are processed to form two-dimensional images, also known as sonogram. This two-dimensional anatomical maps are called B-mode (brightness) images [2].

In principle, US images provide information about internal tissue structures which resulted from interaction between anatomical tissues with the transmitted ultrasound pulse. Due to interaction between ultrasound waves with tissue, backscattered echo signals are produced, in the form of reflection, scattering, interference and absorption. These echo, resulted from coherent summation of ultrasound scatterers, carry information about the tissue under investigation. The nature of coherent summation of such signals gives rise to an interference pattern known as speckle [3].

The despeckling techniques applied in US and SAR imagery can be classified into four main groups, namely, linear and non-linear filters, adaptive speckle filters, wavelet-based filters and anisotropic diffusion-based (AD) approach. In linear filtering technique [4, 5], the multiplicative speckle noise is first converted into an additive noise by applying logarithmic transformation to the speckled image followed by a Wiener filter in order to reject the resultant additive noise. The despeckled image is fully recovered by applying exponential transformation onto the output of Wiener filter. The technique, which convert the multiplicative speckle noise into an additive one, are commonly referred as homomorphic despeckling methods. The Wiener filter is the oldest approach to image denoising, is optimal in the sense of minimum mean-square error (MSE) and is space invariant linear estimator of the signal for images degraded by additive white noise.

The nonlinear filters are possible alternative to the standard linear filters, and the most popular one is the median filter. It has the advantage of preserving edges and is very effective at removing impulsive noise. The median filter sorts the intensities in the neighbourhood window of the reference pixel and calculates the median value of the sorted data. The denoised pixel is obtained by replacing the original reference pixel value by the median value calculated for the particular neigbourhood window [6–8]. The main problem is that the median filter would blur edges and tiny details.

Wavelet-based denoising techniques continue to generate great interest among the computer vision and image processing community. Some of the proposed wavelet-based speckle filters are presented in [9–15]. The success of the technique is due to the fact that in the wavelet domain, the noise is uniformly spread throughout the coefficients, while most of the image information is concentrated in few significant ones. In other word, the wavelet-transformed images tend to be sparse and consequently, noise removal can be achieved by properly suppressing or *thresholding* the small coefficients that are likely due to noise. The wavelet-based denoising techniques involve three major steps, 1) perform a 2-D wavelet transform, 2) modify the noisy coefficients using a shrinkage function, and 3) perform a 2-D inverse wavelet transform [16, 17]. In general, the most critical step in wavelet denoising techniques is the modification of wavelet coefficients. The classification of the different type of wavelet denoising is typically based on it different approach in modifying the noisy coefficients.

The adaptive speckle reducing filters such as Lee, Kuan and Frost can be applicable to both US and SAR images. The methods are developed based on multiplicative model of speckle noise. The methods are based on two assumptions, 1) the recorded image and the speckle noise are statistical independence [18], and 2) a constant ratio of noise standard deviation to mean throughout the image. The second assumption is valid in homogeneous regions. Each of these filters achieved speckle reduction via spatial filtering in a square-moving window known as kernel. The filtering is based on the statistical relationship between the centre pixel and its surrounding pixels within a processing window. The typical window size are 3×3, 5×5, and 7×7. With the window-based techniques, the selection of window will greatly affects the quality of the processed image. If the window is too small, the noise filtering algorithm is not effective, where as if the window is too large, subtle details of the image will be lost in the filtering process.

The squeeze box filter (SBF) which can be classified as an iterative technique, reduces speckle noise by suppressing outliers as a local mean of its neighborhood [19, 20]. Based on the fact that speckle is a stochastic process where outliers inevitably occurs, the proposed SBF achieves noise reduction by iteratively removes the outliers. Specifically, the image pixel outliers are defined to be local minimums and local maximums determined from a 3×3 window. Each outlier will be replaced by a local mean determined from a window centered on the outlying pixel. The outlier pixel value is not used in computing the local mean. After all the outliers are replaced by the local means, the process is repeated until a predetermined number of iteration is reached or until convergence is attained. In [19], experimental results showed that the SBF improves the image quality in terms of contrast enhancement, structural similarity and segmentation result. Although an effective speckle reduction, the SBF however still has artifacts in the form of blurred edges and irregular intensity pattern around edges [21].

In this paper, a subspace-based technique to reduce the speckle noise in US images, is proposed. Fundamentally, the proposed technique is an extension of the original work of Ephraim and Van Trees [22], in speech enhancement towards 2-dimensional signals. The underlying principle is to decompose the vector space of the noisy image into a signal-plus-noise subspace and the noise subspace. The noise removal is achieved by nulling the noise subspace and controlling the noise distribution in the signal subspace. For white noise, the subspace decomposition can theoretically be performed by applying the Karhunen-Loeve transform (KLT) to the noisy image. Linear estimator of the clean image is performed by minimizing image distortion while maintaining the residual noise energy below some given threshold. For colored noise, a prewhitening approach prior to KLT transform, or a generalized subspace for simultaneous diagonalization of the clean and noise covariance matrices, can be used. The fundamental signal and noise model for subspace methods is additive noise uncorrelated with the signal. But, in US images the noise is multiplicative in nature, so a homomorphic framework takes advantage of logarithmic transformation, in order to convert multiplicative noise into additive noise.

The paper is organized as follows. Firstly, the statistic of speckle noise in US images is described. Secondly, the principle of subspace and how it can be extended to speckle noise removal is presented. In specific, this second section covers the proposed subspace technique and its implementation in speckle noise filtering followed by experimental results to determine optimum value of Lagrange multiplier. The subsequent section presents the experimental results to validate and evaluate the performance of the proposed filter. The performance evaluation of the proposed technique is divided into three main categories, 1) using simulated B-mode US images 2) using Field II generated images and 3)using real US images in comparison to Lee filter, wavelet filter [23, 24] in homomorphic framework and SBF technique [19]. The final section concludes this paper.

For clarity, an attempt has been made to adhere to a standard notational convention. Lower case boldface characters will generally refer to vectors. Upper case characters will generally refer to matrices. Vector or matrix transposition will be denoted using (.)^*T*^ and  denotes the real vector space of *m*×*m* dimensions.

## Signal and noise model in ultrasound images

Consider matrix *G* to be the noisy observation of the original image, *W*. Let denote *ξ*_*m*_ and *ξ*_*a*_ as the set of corrupting multiplicative and additive speckle noise components, respectively. The noisy US image can be expressed as [4, 9, 11, 25]
1

Generally, in medical US images, the effect of the additive speckle noise (such as sensor noise) is considerably less significant than the multiplicative component [4, 9, 11, 25]. Taking the assumption that the speckle is fully developed and the additive term can be neglected, equation (1) can be expressed as
2

Applying the logarithmic function to both side of (2), we get
3

Expression (3) can be rewritten as
4

where *Y*,*X* and *N* are the logarithms of *G*,*W* and *ξ*_*m*_ respectively.

The statistical theory to describe US speckle are drawn from the literature of laser optic by Goodman in [26]. Goodman mathematically models speckle as an accumulation of a large number of complex phasors *z*, to be denoted as *z*=*a*+*jb*, also known as complex random walk. These complex phasors, *z* can have either constructive or destructive relationship with each other. Applying central limit theorem to the random walk will results in a signal having two-dimensional Gaussian probability density function (PDF) in the complex plane,
5

where *v*^2^ is the variance of the Gaussian distributed in-phase/quadrature (IQ) components. Equation (5) is simply the product of two independent Gaussian density functions with zero mean and variance *v*^2^ and referred to as a circular Gaussian probability density function. Using the law of conservation of probability, the PDF of speckle phasors magnitude,  is given by
6

For the intensity format, *I*=*A*^2^, the PDF is given by [27]
7

**Figure 1:**
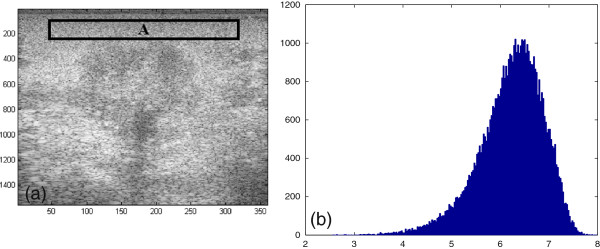
**An US image (a) and histogram of the homogeneous region A (b).**

The equation in (6) and (7) are respectively, known as Rayleigh PDF and exponentional PDF. In B-mode US signal, the magnitude *A* is the quantity of interest since the image is form using envelope detection, in which the phase components are removed. The histogram of the pixels in homogeneous area marked as “A” is shown in Figure 1 which shows a distribution consistent with Rayleigh distribution.

## The subspace-based techniques for noise reduction

In this section, we derive the linear spatial-domain constraint (SDC) estimator, which minimizes the image distortion while constraining the energy of residual noise. The fundamental principle is to decompose the vector space of the noisy image into a signal subspace and noise subspace. The decomposition of the space into two subspaces can be done using either the singular value decomposition (SVD) or the eigenvalue decomposition (EVD). The noise removal is achieved by nulling the noise subspace and controlling the noise distribution in the signal (signal + noise) subspace. We begin with derivation of time (spatial) domain constraints estimator which minimizes the image distortion while constraining the energy of residual noise. Using the signal *X* and an additive noise model *N*, the noisy image matrix can be expressed as *Y*=*X*+*N*. In this case, the error signal *ε* obtained from the linear estimation,  is given by
8

where *ε*_*X*_ represents the image distortion, and *ε*_*N*_ represents the residual noise [22]. Defining the energy of the image distortion , and the energy of the residual noise  as
910

where *E*[·] is the expected value, the optimum linear estimator can be obtained by solving the following spatial-domain constrained optimization problem [22, 28]
11

where *σ* is a positive constant.

The optimum estimator is the sense of Eq. (11) can be found using the Kuhn-Tucker necessary conditions for constrained minimization [29]. It involves solving a constrained minimization problem by applying the method of Lagrange multipliers [30]. Specifically, *H* is a stationary feasible point, if it satisfies the gradient equation of the Lagrangian,
12

where *λ*≥0 is the Lagrange multiplier, and
13

The solution to Eq. 12 is a stationary feasible point that satisfies the gradient equation, ∇_*H*_*L*(*H*,*λ*)=0, thus we obtain
14

thus,
15

Since the noise is assumed to be white, then  where  is the noise variance and *I* is the identity matrix. Hence, the solution for the optimum estimator *H*_*SDC*_ is given as
16

Before the final form of the optimal estimator *H*_*SDC*_ is considered, it is worthy to note that there is a strong empirical evidence indicating that the transformed covariance matrix of most images by the eigenvectors of the *R*_*X*_ have some eigenvalues small enough to be considered as zeros. This means that the number of basis vectors for the pure image is smaller than the dimension of its vectors.

**Figure 2:**
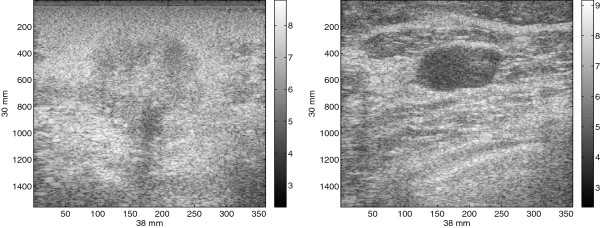
**Uncropped B-mode ultrasound images of breast tissue, malignant tumor (left) and benign tumor (right).** *Courtesy of Ultrasonic Imaging Laboratory at University of Illinois at Urbana-Champaign*.

To verify this key statement, we plot the eigenvalues of two ultrasound images of captured from a patient, as shown in Figure 2. The images shown in Figure 2 correspond to malignant and benign tumor obtained from biopsy-verified studies. The image size is 1556×360 pixels where the x-axis giving the lateral sizes and the y-axis giving the axial sizes. Specifically, for the malignant tumor, the patient was diagnosed with IDC (Invasive Ductal Carcinoma) and for the benign tumor, the patient was diagnosed with fibroadenoma. The RF frames are recorded at 17 frame/second and a total of 12 seconds of data are acquired using a linear transducer array from the Antares®; System. In order to obtain the B-mode ultrasound images, the URI Offline Processing Tools (URI-OPT) run on MATLAB platform is used to convert the RF data to the B-mode images as shown in Figure 2.

**Figure 3:**
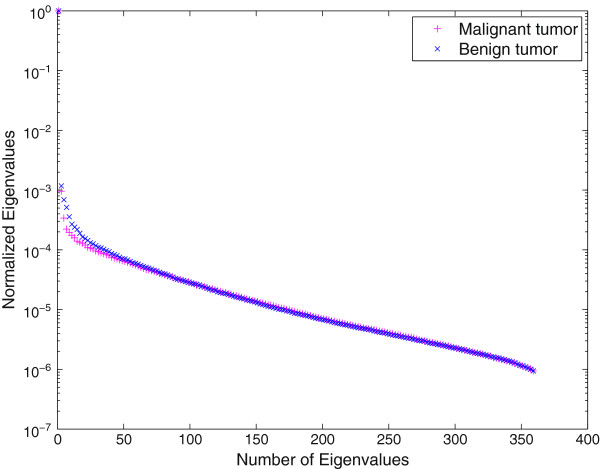
**Eigenvalue profile of** ***R***_***X***_ **, generated from the US images in Figure** 2 **.**

The eigenvalue plot in Figure 3, it shows that some of the eigenvalues of matrix *R*_*X*_ are close to zero, which indicates that the energy of the clean image is distributed among a subset of its coordinates and the signal is confined to a subspace of the noisy Euclidean space. Since all noise eigenvalues are strictly positive, the noise fills in the entire vector space of the noisy image. In other word, the vector space of the noisy image is composed of a signal-plus-noise subspace and a complementary noise subspace. The signal-plus-noise subspace or simply the signal subspace comprises vectors of the clean image as well as of the noise process. The noise subspace contains vectors of the noise process only. Using eigendecomposition of *R*_*X*_=*UΔ*_*X*_*U*^*T*^, Eq. (16) can be expressed as
17

The link between the maximal oriented energy and the signal subspace as well as between the minimal energy and the noise subspace were established in [31]. Using the eigendecomposition analysis [31], in which the , we can improve the form of model matrix *H*_*SDC*_ in Eq. (17) by removing the noise subspace and estimating the clean image from the remaining principal signal subspace
18

In the implementation of SDC, a proper selection of signal subspace dimension *r* and Lagrangian multiplier, *λ* are critical in order to achieve the best noise reduction technique. For subspace dimension, a method based on eigenvalues is proposed in [31, 32] whereas the Lagrangian multiplier is to be empirically determined. As with any other noise filtering technique, the value noise variance needs to be estimated. In this case, the noise variance can be estimated using the last trailing end of the smallest singular value as outlined in [31].

When dealing with ultrasound data, the SDC is implemented in homomorphic framework where the noisy image is first log-transformed prior to SDC filtering. This transformation will convert the multiplicative nature of the speckle to an additive on. The final form of the despeckled image is recovered by performing antilog on the output of the SDC filter. The implementation detail of SDC are given as follows,

1. Apply the homomorphic transformation to the noisy image, *Y*= log(*G*).
2. Estimate the noise variance, .
3. Compute the dimension of signal subspace, *r*.
4. Using the estimated *r* in step 3, apply eigendecomposition on , then extract the basis vectors of signal subspace *U*_1_, and their related eigenvalues .
5. Select the best value of *λ*, then compute the optimum linear estimator, 19
6. Compute the clean image,
7. Reverse the homomorphic effect by taking the exponential of the  as follows 20

In essence, reversing the homomorphic effect in step 7 converts the logarithmic form of the filtered image to a linear form prior to image display.

### Optimum value of the lagrange multiplier

**Figure 4:**
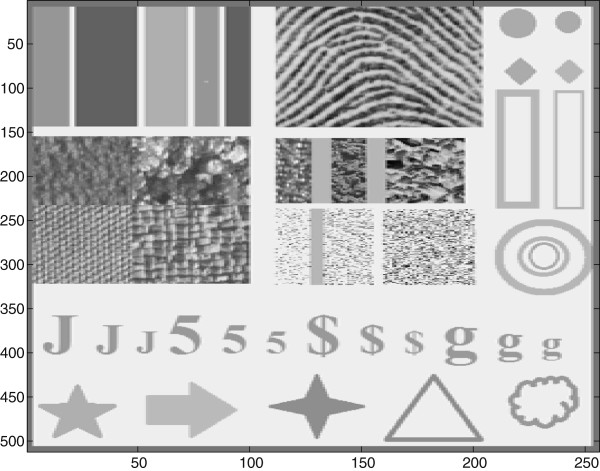
**Test image.**

**Figure 5:**
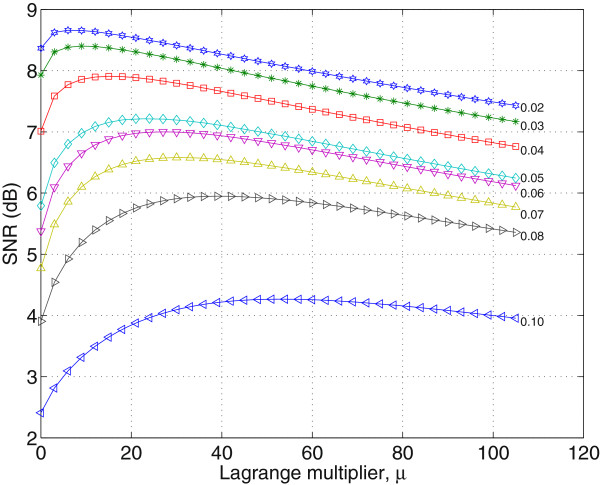
**SNR of the despeckled test image in** 4 **obtained at different** ***λ*** **values.**

To find the best *λ* value for SDC, a test image made up is created as shown in Figure 4. The test image is made up of synthetic patterns, specimens from Broadatz texture set, geometrical shapes, and some alphabets with different size. In particular, the bright and dark strips on the upper left corner closely resemble clinical ultrasound images of carotid artery at the far wall [33]. The test image is selected as it combines different critical features of typical US images. The Broadatz texture is to assess on how well the filter can preserves the texture of the original image. Besides, the different geometrical shapes and alphabets of different sizes are included in order to evaluate the filter capability in preserving edges and fine details of the image. Lastly, the selection of bright and dark strips that closely resemble clinical US images of carotid artery is to assess the filter capability in preserving the artery wall and its edges. The experiment is conducted by corrupting the test image speckle noise of variance extends from 0.03 to 0.05 and *λ* ranging between 1 and 105. The signal-to-noise value (SNR) calculated as
21

where MSE represents the mean-square error, given by
22

is used to indicate the denoising effect of the SDC. The results are shown in Figure 5.

The results in Figure 5 show that the SDC is not too sensitive to the selected value of the Lagrange multiplier. Notably, the results in Figure 5 show that for high noise level, () the despeckle effect of the SDC, measured in terms of the SNR, shows improvement by 1 dB to 1.5 dB, as the Lagrange multiplier varies from 1 to 40. For lower value noise level, () the SNR improvement is around 0.3 dB as the Lagrange multiplier varies from 1 to 10. In general, the results in Figure 5 show better SNR values for higher values of the Lagrange multiplier. However, it should be noted that high value of *λ* may results in oversmoothed images and cause loss of details. Consequently, the rule of selecting *λ* is that for noise variance less than 0.04, *λ* should be selected to be around 10 and with noise variance greater than 0.04 it should be selected to be less than 40.

## Results and discussions

The experimental results presented in this section can be divided into 2 parts. In the first part, the performance of the proposed SDC technique is compared with Lee [34], homomorphic wavelet filter [35] and SBF technique [19] using a simulated speckle image. With a known noise-free image, the performance of SDC is measured in terms of Peak Signal-to-Noise Ratio (PSNR) defined as
23

The value of 255 in Eq. (23) corresponds to the maximum possible pixel value and MSE is defined as in (22).

In the second part, the performance of the proposed SDC technique is investigated using a computer generated image and real US images. Here, the Lee filter is implemented with 7×7 window size, the homomorphic wavelet is used with Daubechies length-eight filter and a 7×7 window and the SBF technique is implemented according to the set up given in [19]. The SDC is implemented as in The subspace-based techniques for noise reduction section. The rank values and the noise variance of the different images are calculated using the method outlined in [31]. As for the Lagrange multiplier, the value is selected using the rule set in the previous section.

When using computer generated US or real US images, the noise-free image is not available which is the practical scenario of denoising applications of US images. Therefore, reference-free methods are used to quantitatively assess the denoising performance. The reference-free methods in this work are mean preservation, normalized variance, autocorrelation [36] and USDSAI [37]. Details on each assessment metric are as follows;

1. *Mean Preservation*: A good speckle filter will maintains the mean intensity within a homogenous region.
2. *Normalized Variance*: The normalized variance indicates the performance of the filter in homogeneous areas. This metric is given by 24

where  corresponds to the mean value of the pixel. In general, lower normalized variance values in the filtered image indicate better speckle suppression.

3. *Autocorrelation*: is another method of filter assessment in homogeneous area where close autocorrelation profile to the original image indicates better texture preservation. The autocorrelation for *m*×*n* image *X* is given as [36] 25

where *X*(*i*,*j*) is the grey value of pixel (*i*,*j*).

4. *Ultrasound Despeckling Assessment Index* (USDSAI): is a modified Fisher discriminant contrast metric [37]. USDSAI gives an indication on how well a despeckling algorithm reduces variances in homogeneous classes while keeping the distinct classes well separated. The metric is defined as 26

where |*C*_*k*_| denotes the number of pixels in class *C*_*k*_. If a despeckling filter produces classes that are well separated then the numerator in 26 will be large. Conversely, if the intraclass variance is reduced, then the denominator will be small giving large value of USDSAI indicating desirable image restoration and enhancement.

### Evaluation of SDC performance in simulated speckle noise scenario

In this experiment, the capability of the SDC technique in reducing the speckle noise is tested and compared with Lee, homomorphic wavelet and SBF technique. The performances of the noise reduction techniques are measured in terms of PSNR values as tabulated in Table 1.

**Table 1:** **PSNR (in dB) values for despeckling of the test image in Figure** 4

Noise variance	Noisy	Lee	Wavelet	SBF	SDC
0.02	20.61	19.88	21.66	21.68	21.73
0.04	18.98	19.73	20.56	20.49	20.60
0.06	16.80	19.46	18.18	19.05	19.84
0.08	14.61	18.96	15.61	18.23	19.11
0.10	11.69	18.07	12.26	17.81	17.67

**Figure 6:**
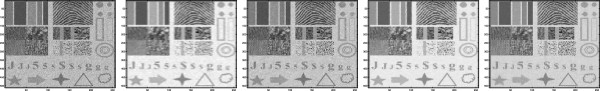
**Restoration of test image in Figure** 4 **at noise variance,**  **.** From left to right, Original, Lee filter, Wavelet filter, SBF and SDC filter.

The results in Table 1 show clearly the better reduction of noise achieved by SDC to Lee, Wavelet and SBF as the noise variance as the noise varies from 0.02 to 0.1. In average, the PSNR value of the SDC is improved by more 3dB followed by SBF(2.9dB), Lee (2.68dB) and Wavelet (1.1dB). However, in order to gain more insight into the performances of the SDC, the denoised images of Figure 4 by SDC, Lee, Wavelet and SBF are shown in Figure 6. Visual inspection of the denoised image by Lee in Figure 6 clearly shows the blurring effect of Lee filter. The wavelet on the other hand shows very close performance to the SDC except for some ringing effect which is visible in the homogeneous part of the image. The SBF exhibits some blurred edges with some noise are not removed around edges. In summary, SDC shows better noise reduction capability and less blurring effect in comparison to Lee and SBF and comparable performance to Wavelet, but with significantly less artifacts and better details preservations.

### Evaluation of SDC performance using a Field II simulated image

**Figure 7:**
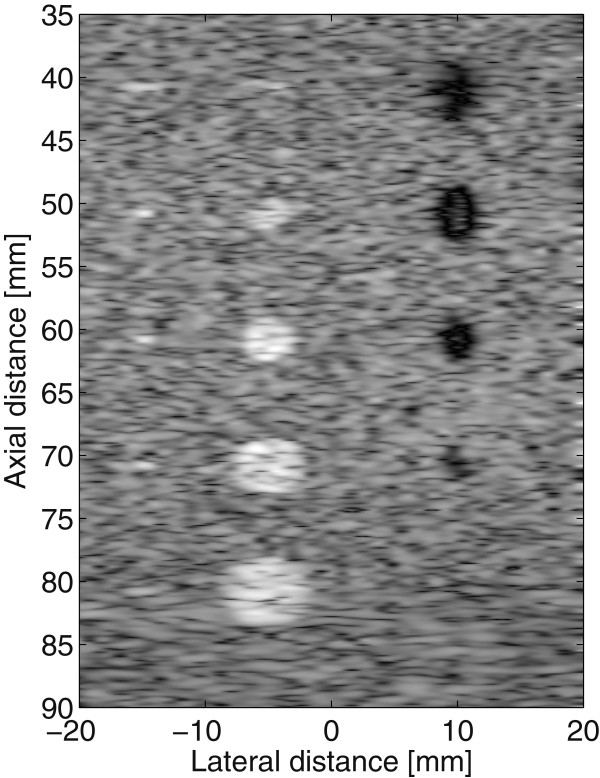
**Uncropped US image of a computer generated cyst phantom.**

In this experiment, the computer model of a cyst phantom is generated using the MATLAB Field II simulation [38, 39]. The phantom contains five point targets; 6, 5, 4, 3, 2 mm diameter waterfilled cysts, and 6, 5, 4, 3, 2 mm diameter high scattering regions. The resulted B-mode US image is shown in Figure 7. The “Cyst” phantom in Figure 7 is composed of 3 constant classes and the filters ability to reduce speckle noise while keeping the distinct classes well separated will be evaluated using normalized variance, mean preservation, preservation of autocorrelation [31] and USDSAI assessment metric. Prior to despeckling, the cyst image is converted into an 8-bit image of size 512×512 pixels.

**Figure 8:**
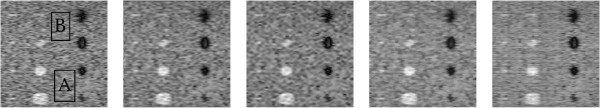
**Restoration of cyst image generated from Field II simulation.** From left to right, Original, Lee filter, Wavelet filter, SBF and SDC filter.

In the first experiment, the normalized variance and mean preservation for the cyst image are calculated over two selected regions labeled as A and B as in Figure 8. The normalized variances of the two regions calculated before and after denoising for SDC, Lee, Wavelet and SBF are presented in Table 2. The results in Table 2 show clearly the better reduction of noise achieved by SDC compared to Lee, Wavelet and SBF over the two homogeneous regions. In order to further verify the better better performance by the SDC, the denoised images of Figure 7 by SDC, Lee, Wavelet and SBF are shown in Figure 8. Visual inspection of the denoised images in Figure 8 clearly shows far less introduced blurring effect, better noise reduction, and better contrast enhancements by the SDC in comparison to the Lee, Wavelet and SBF. On the other hand, Figure 8 also shows that the SBF introduces relatively similar blurring effect to Lee and Wavelet though it gives better contrast enhancement values, measured in terms of USDSAI as tabulated in Table 3.

**Table 2:** **Normalized variance in denoised images of the cyst phantom in Figure** 8

	Original	Lee	Wavelet	SBF	SDC
Region A	0.03	0.02	0.02	0.02	0.01
Region B	0.04	0.02	0.02	0.01	0.01

**Table 3:** **USDSAI value in denoised images of the cyst phantom in Figure** 8

Original	Lee	Wavelet	SBF	SDC
1.00	2.10	1.80	3.07	3.00

In addition to variance reduction, the values of mean preservation for the two regions calculated before and after denoising for SDC, Lee, Wavelet and SBF filter are also evaluated and included in Table 4. The results in Table 4 indicate the better capability by Lee to Wavelet, SBF and SDC in preserving the mean value in the computer generated cyst image in Figure 7. The better mean preservation by Lee is highly expected because of the averaging scheme of Lee filter which tends to maintain the mean value in the image.

**Figure 9:**
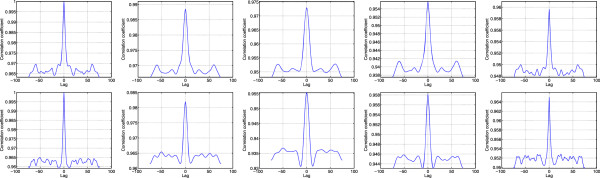
**Autocorrelation profile for Region A (top) and Region B (bottom) of cyst image in Figure** 7 **.** From left to right, Original, Lee filter, Wavelet filter, SBF and SDC filter.

In order to assess the capability of the different algorithms in texture preservation in the denoised image, the autocorrelation in region A and B of the cyst image in Figure 7 are calculated before and after speckle filtering and depicted in Figure 9. The autocorrelation profiles in Figure 9 clearly show the better details preservation by the SDC in comparison to Lee, Wavelet and SBF. Notably, the profiles by Lee, Wavelet and SBF exhibit wider profiles in the neighbourhood of zero lag and largely deviated from the original at other lags. On the contrary, the SDC shows close autocorrelation profile of the denoised image to the original one in terms of shape and better preservation of the unit impulse structure at zero lag value than Lee, Wavelet and SBF.

**Table 4:** **Mean preservation in denoised images of the cyst phantom in Figure** 8

	Original	Lee	Wavelet	SBF	SDC
Region A	127.74	127.87	126.73	134.87	126.48
Region B	125.60	125.69	123.80	133.02	125.17

### Evaluation of SDC performance using real US images

In this experiment, the performance of the proposed SDC is analyzed and compared with Lee and Wavelet using ultrasound images captured from a patient as shown in Figure 2. The images are biopsy-verified studies and presented with non-palpable tumors initially detected by mammography [40]. These images are shown in Figure 2 for malignant and benign tumor. In Figure 2, the patient with malignant tumor was diagnosed with invasive ductal carcinoma whereas the patient with benign tumor was diagnosed with fibroadenoma. The image size is 1536×256 pixels with the x-axis and the y-axis giving lateral sizes and axial sizes of the image, respectively. The RF frames are recorded at 17 frame/second and a total of 12 seconds of data are acquired using a linear transducer array from the Antares^®;^ System. In order to obtain the B-mode ultrasound images, the URI Offline Processing Tools (URI-OPT) run on MATLAB platform is used to convert the RF data to the B-mode images as shown in Figure 2.

**Figure 10:**
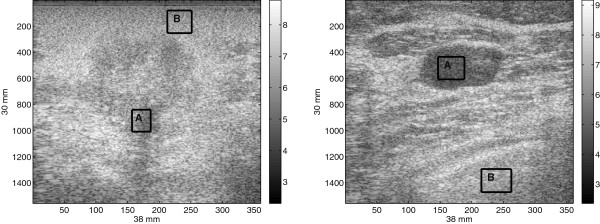
**Region A and B in the US images of the breast tissue of Figure** 2 **.**

**Figure 11:**
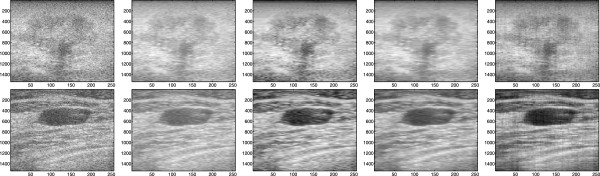
**Restoration of malignant tumor (top) and benign tumor (bottom) in Figure** 2 **.** From left to right, Original, Lee filter, Wavelet filter, SBF and SDC filter.

In the first part of this experiment, two homogeneous areas are selected and marked as region A and B Figure 10. In order to assess the capability of the filters in reducing noise in image, variances are calculated over these two regions before and after denoising the image in Figure 2. The values of normalized variance are tabulated in Table 5. The results in Table 5 indicate the better noise reduction capability by the Wavelet in comparison to Lee, SBF and SDC which show a relatively comparable performance. However, in order to gain more insight into the performance of the Wavelet and to aid the interpretation of the results in Table 5, the denoised images by Lee, Wavelet, SBF and SDC are shown in Figure 11. The results in Figure 11 clearly show that the main reason for the high noise reduction values by the Wavelet in Table 5 is the intensive appearance of wavelet artifacts in its denoised image. On the other hand, though the SDC gives approximately similar values to Lee and SBF in Table 5, the denoised images in Figure 11 show clearly better noise reduction and image details preservation.

**Table 5:** **Normalized noise variance in the denoised images of real US images in Figure** 2

Malignant tumor	Original	Lee	Wavelet	SBF	SDC
Region A	0.012	0.003	0.001	0.003	0.003
Region B	0.009	0.004	0.001	0.004	0.003
**Benign tumor**	**Original**	**Lee**	**Wavelet**	**SBF**	**SDC**
Region A	0.015	0.004	0.001	0.004	0.004
Region B	0.018	0.007	0.003	0.007	0.005

In addition to the noise reduction capability addressed by the normalized variance, the mean preservation capability is also tested and presented in Table 6. The results show the better performance of Lee in preserving mean value and this performance is very close to SDC. Notably, the mean value of Lee and SDC only differs by no more than 0.03. On the other hand, the result on Wavelet and SBF indicates poor preservation of mean by the two filters. In terms of contrast enhancement, given by the USDSAI values as shown in Table 7, the SDC gives better contrast enhancement to both Lee and Wavelet but a comparable performance to SBF.

**Figure 12:**
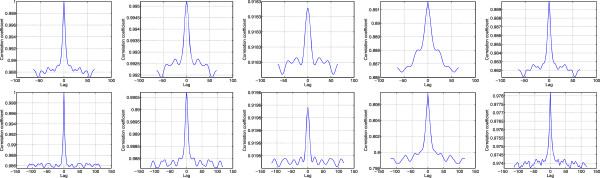
**Autocorrelation profile for Region A of malignant tumor (top) and benign tumor (bottom) in Figure** 2 **.** From left to right Original, Lee filter, Wavelet filter, SBF and SDC filter.

To gain more insight into the performance of the three considered techniques, their capability in preserving the characteristics of the original image is tested in terms of autocorrelation profiles of the selected region, A and B as shown in Figure 12. The results in Figure 12 give clear indication on the better preservation of the texture of the original image by SDC in comparison to Lee, Wavelet and SBF. In fact, the SDC shows close autocorrelation profile of the denoised image to the original one especially in term of shape and better preservation of the unit impulse structure at zero lag than Lee, Wavelet and SBF. Moreover, the autocorrelation profiles produced by Lee and Wavelet shows widened profiles at zero lag and largely deviated profiles from the original at other lags.

**Table 6:** **Mean preservation in the denoised images of real US images in Figure** 2

Malignant tumor	Original	Lee	Wavelet	SBF	SDC
Region A	5.29	5.30	0.72	12.80	5.29
Region B	7.49	7.49	0.87	14.90	7.46
**Benign tumor**	**Original**	**Lee**	**Wavelet**	**SBF**	**SDC**
Region A	4.83	4.84	0.68	12.32	4.83
Region B	5.23	5.24	0.72	12.75	5.24

**Table 7:** **USDSAI value in denoised images of real US images in Figure** 2

	Original	Lee	Wavelet	SBF	SDC
Malignant	1.00	2.63	2.96	4.11	4.09
Benign	1.00	2.83	2.70	4.22	4.17

In the third experiment, the required computational time by Lee, Wavelet, SBF and SDC to process the ultrasound images of Figure 2 are calculated and included in Table 8. The filters are implemented on MATLAB platform using a computer with Intel(R) Xeon(R) 5607 @ 2.27 GHz processor and 8GB RAM. The results in Table 8 shows that the computational times of both SDC and wavelet are almost similar and less by nearly 3 times SBF and 10 times than Lee (7 × 7).

**Table 8:** **Computational time (in second) of Lee, Wavelet, SBF and SDC for the US image in Figure** 2

	Lee	Wavelet	SBF	SDC
Benign	63.97	8.88	17.46	6.20
Malignant	63.57	8.77	20.52	6.30

## Conclusions

A subspace-based denoising technique for US images is presented and tested. The proposed technique, SDC is based on linear estimator and rank reduced subspace model to estimate the clean image from the corrupted one with speckle noise. The performance of the SDC is tested with simulated and real data, and compared with Lee and wavelet. The results indicate better noise variance reduction capability with the simulated images by the SDC than Lee, Wavelet and SBF in addition to less blurry effect. With the real case scenario, the SDC, Lee, Wavelet and SBF are tested with images obtained from raw RF data. The performances are calculated in terms of noise reduction, improvement in image contrast and preservation of the autocorrelation profiles. The results indicate that SDC offer better texture preservation, measured in terms of autocorrelation profiles and good contrast enhancement, measure in terms of USDSAI value. Finally, the computational complexity of the algorithms is compared and the results show that SDC required the least computational time compared to Lee, Wavelet and SBF.
